# Fringe Capacitance Correction for a Coaxial Soil Cell

**DOI:** 10.3390/s110100757

**Published:** 2011-01-12

**Authors:** Mathew G. Pelletier, Joseph A. Viera, Robert C. Schwartz, Robert J. Lascano, Steven R. Evett, Tim R. Green, John D. Wanjura, Greg A. Holt

**Affiliations:** 1 Cotton Production and Processing Unit, USDA-ARS, Lubbock, TX 79403, USA; 2 Sensors Group Microsemi Corporation Lowell, MA 01851, USA; 3 Soil and Water Management Research Unit, USDA-ARS, Bushland, TX 79012, USA; 4 Agricultural Systems Research Unit, USDA-ARS, Fort Collins, CO 80526, USA; 5 Wind Erosion and Water Conservation Unit, USDA-ARS, Lubbock, TX 79403, USA

**Keywords:** TDR, cotton moisture, moisture sensing, permittivity, microwave sensing, microwave moisture

## Abstract

Accurate measurement of moisture content is a prime requirement in hydrological, geophysical and biogeochemical research as well as for material characterization and process control. Within these areas, accurate measurements of the surface area and bound water content is becoming increasingly important for providing answers to many fundamental questions ranging from characterization of cotton fiber maturity, to accurate characterization of soil water content in soil water conservation research to bio-plant water utilization to chemical reactions and diffusions of ionic species across membranes in cells as well as in the dense suspensions that occur in surface films. One promising technique to address the increasing demands for higher accuracy water content measurements is utilization of electrical permittivity characterization of materials. This technique has enjoyed a strong following in the soil-science and geological community through measurements of apparent permittivity via time-domain-reflectometry (TDR) as well in many process control applications. Recent research however, is indicating a need to increase the accuracy beyond that available from traditional TDR. The most logical pathway then becomes a transition from TDR based measurements to network analyzer measurements of absolute permittivity that will remove the adverse effects that high surface area soils and conductivity impart onto the measurements of apparent permittivity in traditional TDR applications.

This research examines an observed experimental error for the coaxial probe, from which the modern TDR probe originated, which is hypothesized to be due to fringe capacitance. The research provides an experimental and theoretical basis for the cause of the error and provides a technique by which to correct the system to remove this source of error. To test this theory, a Poisson model of a coaxial cell was formulated to calculate the effective theoretical extra length caused by the fringe capacitance which is then used to correct the experimental results such that experimental measurements utilizing differing coaxial cell diameters and probe lengths, upon correction with the Poisson model derived correction factor, all produce the same results thereby lending support and for an augmented measurement technique for measurement of absolute permittivity.

## 1. Introduction

Frequency domain analysis of soils, cotton lint, biological cells and media is rapidly gaining appreciation due to the ability to provide a true measurement of permittivity as opposed to an apparent permittivity that TDR analysis in the time domain provides. One of the driving factors behind this new trend is due to the recognition that in saline and high clay content soils, that the conductive soils dielectric loss has a profound impact on the measured apparent permittivity which causes large errors especially when temperature effects are taken into consideration.

Recent research [1], reports the use of frequency domain analysis for extending the use of TDR waveforms in conductive soils as an alternative solution to soils in which the standard TDR waveform return is lost due to excessive conductivity, which renders the traditional TDR technique unusable or highly inaccurate. In this report, they report the need for use of a correction equation to relate the measured scattering S11 parameters to the soil dielectric properties, was suggested by Clarkson [2]. Other researchers have also reporting success in the use of the Clarkson [2] correction Equations [3–5]. Of note was a report by Hoekstra and Delaney [6], that warned of possible additional modes of propagation, *i.e.*, TE and TM propagation modes, that they proposed may cause an error in higher frequencies than the Clarkson [2] formulation wouldn’t address as the Clarkson [2] equation assumes TEM is the only mode of propagation in the coaxial or TDR cell. Additional similar research was reported [4], which hypothesized that additional propagation modes were a likely cause of perceived errors in their higher frequency measurements from their expected theoretical responses.

Another similar research path [7], utilized an impedance calculation of a transmission line terminated with an open ended coaxial soil-filled cell, which was derived along an alternative formulation linking measured reflection spectral response to the permittivity parameters, thereby providing a separate path to the correction of the measured spectrum to that of a free space plane wave propagation. This formulation has become popular of late and has been used with slight modifications by several researchers [8–11].

In comparing the two approaches taken by Clarkson and Kraft [2,7], of note is that they both used as their basis a transmission line terminated in a simple coaxial soil-filled cell. However, neither of the above mentioned formulations provide a means of correction for the other system components, *i.e.*, cable, cable length, connectors, multiplexors and instrument effects such as instrument to cable impedance miss-match, non-ideal pulse, time varying pulse ect. One example of the fundamental need for such corrections are provided in reports of the effects of exterior equipment such as variations in coaxial cable lengths, transient suppressors ect., ([4,12,13], on the obtained measurements). Further evidence is provided by Jones and Or [1] and Freil and Or [5], by their encouragement to utilize permittivity standards by which to judge obtained measurements against known standards. In moving toward utilization of permittivity standards, of critical need are calibration methods that couple models such as Clarkson [2] and Kraft [7] to high quality calibration methods such as are utilized in the microwave engineering field for use in Network Analyzer measurements [7,16,17]. In moving forward towards resolving these issues, this research examines the terminated coaxial cell from a theoretical basis to provide a sound background by which to examine an observed experimental error which is hypothesized to be due fringe capacitance. Of particular importance, we will show that the magnitude of this error is such that for measurements of low permittivities, such as for moisture measurements in loose cotton, for measurements of water contamination in oil, or in measurements where the permittivity is under-going small changes, this error is significant and leads to large errors in the measured permittivity if not corrected for. This research then applies the developed theory towards confirmation via experimentation and finally provides a solution for the removal of this error from the measurement.

Objectives: Derive a technique for the absolute measurement of permittivity from coaxial cells and,

- show the impact of fringe capacitance on experimental measurements,
- develop a theoretical model and predict the magnitude of the hypothesized error due to fringe- capacitance,
- provide experimental results that quantify the magnitude of the fringe capacitance error,
- show the correlation between the predicted error to the experimental error,
- develop a correction solution for removal of this error from coaxial and TDR probe measurements.

**Theory:** As the research community moves towards higher accuracy demands on TDR measurements, the natural evolution of the science will be to transition toward Network Analyzer measurements in the frequency domain, due to the significant improvement in the accuracy and dynamic range the Network Analyzer provides over the traditional time domain TDR measurements, as well as the ability to utilize absolute permittivity standards which then enhances the accuracy of transferability of data between researchers. In moving from a measurement of apparent permittivity in the time domain towards a measurement of true permittivity and loss in the frequency domain, the response of the TDR or coaxial probe must be removed from the measurement. The following section details the electrodynamics to form a frequency response characterization for later removal of confounding affects to obtain a measurement of the true permittivity of the soil.

*Formulation of frequency response of terminated open-ended coaxial probe*. As the modern TDR probe is widely accepted as being closely aligned to the coaxial cable, the analysis starts with the formulation for a coaxial cable by which to find the frequency response of the structure.

We note that for propagation of a free-space plane wave, in a source-less region, that is directed only in the z direction, the form of the wave propagation can be shown to have the form of Equation 1, with the propagation coefficient γ as shown in Equation 2, [1].

(1) $$e^{\gamma }=e^{\mathrm{jk}}=e^{\alpha +j\beta }$$

(2) $$\gamma =\mathrm{jk}=\alpha +j\beta =j\sqrt{\varpi^{2}\mu (\varepsilon^{\prime }-j\frac{\sigma }{\varpi }-j\varepsilon^{\prime \prime })}=j\sqrt{\varpi^{2}\mu \varepsilon^{\prime }(1-j\;\text{tan}\;\delta )}$$

Where:

ε′: = real, dielectric constant, term of the complex permittivity (F/m).
ε″: = imaginary, loss, term of the complex permittivity (F/m).
γ: = propagation coefficient (1/m).
α: = attenuation factor of the propagation coefficient (nepers/m).
*j*: = unit imaginary number √-1.
β: = phase delay factor of the propagation coefficient (rads/m).
σ: = conductivity factor of the propagation coefficient (S/m).
μ: = material permeability (H/m).
ω: = omega (rads/s).
e: = the exponential transcendental number = 2.71828…
tan δ: = loss tangent definition as ratio of real to imaginary part of displacement current.

**Figure 1.**
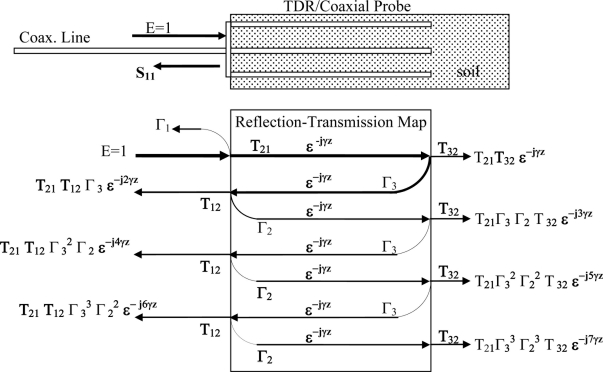
Reflection/transmission map detail of resultant waveform from combination of multiple reflections from both the leading edge, undesired, and probe end, desired measurement, in TDR/FDR probes, or coaxial cells, due to impedance miss-match between inter-connecting coaxial cable’s impedance, of Zo, to the soil-probe impedance of Z1. Note: Hatched area indicates soil or other material under test.

Of note is that while for free-space wave propagation, Equations 1 and 2 are sufficient to provide a means to measure complex permittivity, however when measurements are taken with a TDR or coaxial probe, the probe structure modifies how the plane wave propagates inside the TDR waveguide. Thus, to obtain a measurement of absolute permittivity, rather than apparent permittivity, it will be necessary to also characterize the waveguide’s frequency response effect on the plane-wave in order to extract the real permittivity from a measurement taken with a coaxial probe. We also note that of importance is the impedance miss-match between the coaxial cell and the interconnecting cable as the impedance miss-match sets up multiple reflections, Figure 1, that creates large ripples subsequently create significant errors for measurements in the time and frequency domain which in turn translate to significant errors in the measured permittivity if left uncorrected.

Utilizing a power series closed form solution to an infinite series, provides an exact solution to the measured response of the coaxial cell that is due to the impedance miss-match between the coaxial cable and the interconnecting cable which can be used to model the measured reflection coefficient, Γ_measured_, to that of the desired measurement of the free space propagation constant γ, which is required for determination of the material’s true permittivity as shown in Equation 3, [14,16,18]..

(3) $$\Gamma_{\mathrm{measured}}=\frac{\Gamma_{1}+\Gamma_{3}e^{-2j\gamma z}}{1+\Gamma_{1}\Gamma_{3}e^{-2j\gamma z}}$$

Of particular interest to this research is that even when the measured permittivity is corrected for the multiple reflections, per use of Equation 3, there is still an unaccounted for error, which lead us to the primary focus of this paper.

## 2. Experimental Section

**Figure 2.**
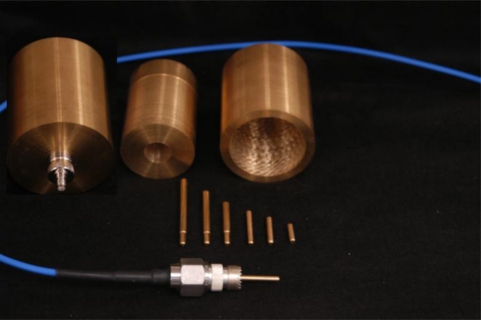
Machined brass coaxial cells based on commercial N to UHF (RF) adapter. System utilizes an insert based center probe that allows for changing the center probe, thereby providing a center-probe length change for the system, while maintaining the original calibration that removes the effects of the instrument, interfacing cable and the RF adapter, while preserving the original system calibration. On the right is the large brass insert that provides a similar means to maintain the original system calibration while providing the means to alter the outer diameter of the coaxial cell, hence altering the impedance, of the coaxial cell. This system was designed with the center insert installed, to provide a near perfect 50 Ω match for the coaxial cell when filled with dry sand ε_r_ = 2.85.

In the interest of obtaining guidance into the levels of expected accuracy that can be obtained by the proposed miss-match impedance correction protocol in a non-radiating condition, provided by Equation 3; experiments were conducted utilizing full coaxial open terminated cells machined out of brass, Figure 2.

Brass was chosen for the coaxial cell as Kraft [7], suggested there may be some additional confounding effects due to the permeability of steel that is known to provide an added permeability loss and hence an expected increase in the experimental errors. To remove the effects of radiation, the maximum length of the probes was selected to limit radiation to frequencies above 3.5 GHz, thus, 4.25 cm was the longest probe utilized in the set. Finally, as Equation 3 indicates that a change in diameter, and its subsequent change in the impedance of the coaxial cell, will cause a change in the obtained measured reflectance coefficient; of interest was the comparison of two otherwise identical coaxial cells with different outer-diameters to adjust the coaxial cell’s effective impedance.

Noting that for accurate utilization of a Network Analyzer, a major requirement is for the instrument and interconnecting cables and connectors to be calibrated out of the system. This calibration requirement however causes some difficulties in performing direct comparisons between the two like-coaxial cells, as ideally one would like to use the same calibration for both cells in order to avoid the calibration from obscuring the impact of the differences. In order to achieve this single calibration/dual use condition, a close fitting drop-in insert, designed to reduce the outer diameter, was machined to allow for direct comparison of two probe geometries without the need for changing connectors and the probe structure, thereby avoiding the need for a recalibration thereby enhancing the accuracy of the comparison.

The outer diameter, 50.67 mm, of the large coaxial cell was chosen for similar dimensions to industry standard 20 cm TDR probes, with a similar center conductor diameter of 4.67 mm. The smaller diameter insert provided a reduced 17.96 mm outer diameter, which was chosen to give a 50 Ω impedance when the coaxial cell was filled with dry sand, with an estimated relative permittivity of ε_r_ = 2.85. Further in an effort to increase the confidence of the obtained results, multiple internal probes, the current carrying member of the inner diameter of the coaxial cell, were all machined at the same diameter at different lengths, for comparison of experimental results to the theoretically predicted values for delays that are a direct function of the path-length provided by the length of the center conductor that is exposed to the material under investigation.

In the interest of restricting the internal reflections to only those of the model of Figure 1, care was taken in the construction of the coaxial cell to coaxial cable interface. The research eventually identified that female UHF to SMA coaxial adapters provide an ideal interface, as the female UHF adapter is threaded with 5/8–25 threads which provides a high-quality coupling to a machined and like-threaded coaxial cell, while providing a commercial quality rf connection between the coaxial cell and the coaxial cable. Upon investigation of the proposed setup of Figure 2, and the TDR results indicated the coaxial cell provides a clean transition for the matched configuration with only a minor low-impedance capacitive reflection occurring mainly inside the UHF to SMA transition which is easily removed from the measurements through a standard short-open-load one-port calibration protocol on the network analyzer.

For the network analyzer calibration, referenced earlier, the research used an open-short-load protocol to move the reference plane to the location of the short. Some experimentation on calibration for this system, lead to the realization that a choice had to be made between using either an in-house built shorting element that provides the short at the correct location, thereby establishing the reference plane correctly; or to utilize a high-quality commercial short, designed specifically for calibrations, that would inadvertently put the calibration plane in the wrong location, thereby leading to a phase error that would have to be corrected via a model. Experiments suggested that for the highest accuracy work, a well designed in-house constructed short made from an identical connector to the one used in manufacturing the coaxial cell, provided the best results.

## 3. Results and Discussion

We note that due to the impedance miss-match issues, when dealing with materials with a wide range of permittivity’s, it becomes very difficult to separate multiple reflection errors from other mitigating factors. However, there is a special resonance frequency that is free from multiple reflection errors that can be used to examine additional errors that are not due to impedance miss-match. The use of this technique, quarter-wave impedance transformer, to remove the impedance miss-match error is what led to the discovery of the additional error that is hypothesized to be created by fringe capacitance emanating off the center conductor’s end-point (tip).

As a first comparison into the validity of the miss-match impedance correction technique, we note the work by Heimovarra [15], which also suggests an accurate measurement can be made without an impedance miss-match at the ¼ wavelength frequency as the impedance at the ¼ wavelength frequency is 50 Ω, hence the measurement is correct a this one frequency that is free from impedance miss-match. This observation is also well accepted in microwave engineering and is utilized in the technique of impedance matching using a quarter-wave length line as an impedance transformation structure, [15,17].

For the case where the system is almost matched at the ¼ wavelength line length, the discontinuities between impedances are small and the product Γ_1_ Γ_3_ ≪ 1, which effectively reduces the denominator in Equation 3 to approach unity, leaving only the numerator of Equation 3 as the approximation. Thus, for small changes in impedances between the cable and the coaxial cell, Equation 3 can be approximated by Equation 4.

(4) $$\Gamma_{\mathrm{measured}}=\Gamma_{1}+\Gamma_{3}e^{-2j\gamma z}$$

Further noting that by definition at the ¼ wavelength the following condition is true (assume very low loss condition).

(5) $$\gamma z=(\alpha +j\beta )z=\frac{\pi }{2}$$

Thus at the ¼ wavelength frequency equation, for the non-radiating conditions *Γ_3_=1*, then Equation 4 reduces to.

(6) $$\Gamma_{\mathrm{measured}}=\Gamma_{1}+e^{-j\pi }=\frac{Z_{1}-Z_{0}}{Z_{1}+Z_{0}}+e^{-j\pi }$$

Noting that in the matched condition,

(7) $$Z_{C}=Z_{0}$$

Which leads to

(8) $$\Gamma_{\mathrm{measured}}=-1∠\pi$$

Thus, when the phase of the reflected wave is delayed by π radians, the frequency of this occurrence corresponds to the ¼ wavelength matched condition. Further noting that at the matched ¼ wavelength condition, Zc is equal to Zo which is equal to the coaxial cell impedance, Equation 9 [16].

(9) $$Z_{C}=Z_{0}=\frac{1}{2\pi }\text{ln}(\frac{b}{a})\sqrt{\frac{\mu_{0}}{\varepsilon }}$$

Re-arranging Equation 9 provides Equation 10.

(10) $$b=a\;{\text{exp}}^{\left(2\pi \varepsilon Z_{0}\sqrt{\frac{1}{\mu_{0}}}\right)}$$

Equation 10 indicates that when the coaxial cell is matched via a ¼ wavelength resonant transformer, for a given ε permittivity, there is only one outer diameter that will provide a matched condition. Thus, for all other ε permittivity’s, the coaxial cell will be miss-matched and will require correction via an inverse solution to Equation 3.

For the purposes of relating this theory to experimentally derived measurements, some basic test cases utilizing low loss materials were investigated. For the low loss, Γ = 0, test cases and for true TEM lines at ¼ wavelength, which occurs at the λ/4 phase delay which indicates the resonant frequency with matched condition, provides a direct measure of the propagation coefficient γ, as shown in Equation 11.

(11) $$\gamma z=\beta z=\frac{\pi }{2}=\beta \left(\frac{\lambda }{4}\right)=\frac{2\pi f}{v_{p}}\left(\frac{\lambda }{4}\right)=2\pi f\frac{\sqrt{\mu_{r}\varepsilon_{r}}}{c}\left(\frac{\lambda }{4}\right)$$

Rearranging 11 provides Equation 12, provides the theoretical low loss resonant frequency for a given probe length whose physical length corresponds to the ¼-λ resonance. Alternatively, the wavelength is equal to 4 times the physical probe length, for a given ε_r_ relative permittivity due to the two wave propagation caused by the open-circuit reflection off the end of the probe.

(12) $$f_{\mathrm{resonance}}=\frac{c}{\lambda \sqrt{\mu_{r}\varepsilon_{r}}}$$

Of particular note, is that Equation 12 is not dependent upon the coaxial cell’s geometry, as noted by Equations 6–8. Furthermore, calculations utilizing Equation 12 to find the location of the ¼ wavelength resonant frequency for the air filled coaxial cell, shows the theory predicts the same location for the smaller inner-diameter coaxial cell as the larger-diameter coaxial cell. However, our experimental results clearly showed a difference in the location of the ¼ wavelength resonant frequency between the smaller diameter coaxial cell to the larger diameter coaxial cell when air or dry sand (low loss material) is placed inside the coaxial cell. It is noticed that the deviation in frequency between the two diameter cells, all other parameters remaining constant, is over 100 MHz, which represents a significant deviation in the length of coaxial probe, or the measured permittivity for low permittivity materials, such as water contamination of oil, or moisture measurements of un-compacted cotton. Thus, there must be some other effect that is not accounted for in the impedance miss-match theory, as presented thus far in this paper. It should be also noted that this large an error, as recorded by the difference in the measured resonant frequency, likely carries over to an error in all frequencies and thereby is expected to affect the accuracy of the obtained permittivity even after correction for impedance miss-match by Equation 3. As an example, at 350 MHz, the measured response with the large diameter coaxial cell was a delay of 42 degrees, however with the reduced diameter of the small coaxial cell, the delay was reduced to only 36 degrees, a difference of 16% in the measured propagation delay. This propagation delay error is the difference in measured permittivity of ε′ = 2.5 from the actual ε′ = 3.3, which in turn would be translated into a significant error in the estimation of the quantity of volumetric moisture in oil or cotton. Thus, for high accuracy absolute permittivity sensing, especially in materials exhibiting low permittivity values, there is a critical need for resolving this issue.

*Fringe Capacitance*: It was hypothesized that this additional error was due to fringe fields emanating off the center conductor’s probe-tip. Thus, additional efforts were expended to explore if the observed experimental deviation from the predictions of the resonant frequency, was due to fringe capacitance between the end of the inner probe to the outer wall of the coaxial cell. Noting from Collin [16] and Pozar [18], the TEM propagation mode in the coaxial cell can be modeled by a quasi-static Poisson Equation 13; a Poisson model was utilized to examine the fringe capacitance concept for error correction of the coaxial cell.

(13) $${\varepsilon \nabla }^{2}V=\rho$$

Where,

ρ := current density
V:= Voltage (electric potential)
∇ := Gradient Operator
ε := Permittivity of medium.

**Figure 3.**
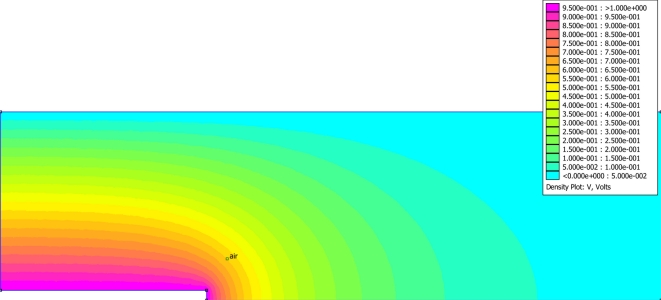
Poisson cross-sectional model of coaxial cell with terminated center conductor, which is the notched area at the bottom of the screen. The color pictorial is of the electric-potential inside a cross-section of a coaxial, or TDR probe with symmetry boundary conditions applied along the bottom edge (not including the notched section which represents the center conductor and has an imposed Voltage). The left and top edge of the figure have a zero voltage boundary condition representing the conductors being tied to ground and the far right edge was set to a floating boundary condition to represent an open-ended coaxial cell in a homogenous media.

Noting from Collin [16] and Pozar [18], the dominant TEM propagation mode in the coaxial cell can be modeled by a quasi-static Poisson Equation 13, which was utilized to examine the fringe capacitance concept for support in the development of an error correction for the coaxial cell, Figure 3.

**Figure 4.**
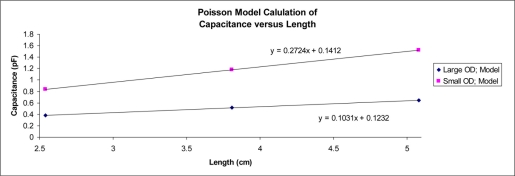
Comparison of the capacitance as calculated from the Poisson cross-sectional model of coaxial cell with terminated center conductor, as a function of the center conductor’s probe length.

To find the fringe capacitance from the Poisson model, our protocol was to find the total capacitance for each simulation run, by means of the relation of cell’s capacitance being equal to the charge on the conductors divided by the voltage between the conductors, C = Q/V. By performing multiple simulations as the length of the center conductor was reduced, the results of several of the models at progressively shorter center conductors provides an estimate of the fringe capacitance which is found from the intercept on the graph of capacitance *versus* center conductor length, (Figure 4). The significant intercept supports the fringe capacitance hypothesis, is creating an artificial extension to the center conductor.

To examine the predicted δ–length, or δγ, experimentally, several center conductors of varying lengths, shown in Figure 2, were constructed and tested. It was found that the predicted capacitance extra-length effect can be duplicated in experiments and then quantified by letting the length of the probe approach to zero, with respect to the end of the coaxial holder that supports the center rod, by subsequent replacement with progressively shorter probes, whereby it is found that the electrical zero location actually occurs well inside the connector, rather than flush with the end of the connector, thereby providing experimental support for the fringe field effect hypothesis.

Given the Poisson model, along with qualitative experimental evidence, suggests a unique length extension δλ is required to correct for the extra fringe capacitance in coaxial cell type measurements, or TDR soil probes; therefore we propose a new variant for Equation 12, to allow for correction of the fringing field effects which is proposed herein as Equation 14.

(14) $$f_{\mathrm{resonance}}=\frac{c}{(\lambda +\delta \lambda )\sqrt{\mu_{r}\varepsilon_{r}}}$$

Where

δλ:= effective wavelength extension of the center probe due to fringe capacitance.

To utlize Equation 14, the magnitude of the length extension δγ can be found by the inversion of Equation 14, into Equation 15.

(15) $$\delta \lambda =\frac{c}{f_{\mathrm{resonance}}\sqrt{\mu_{r}\varepsilon_{r}}}-\lambda =\frac{c}{f_{\mathrm{resonance}}\sqrt{\mu_{r}\varepsilon_{r}}}{-4L}_{\mathrm{probe}}$$

Where:

L_probe_ := physical length of the probe (m).

In practice, it was found that the experimentally derived lengths provided the following estimates for a required length extension when tested with air as the dielectric; small OD cell δλ = 0.25 cm and large OD cell’s δλ = 0.49 cm, that were similar to those predicted by the Poisson model. In practice it is suggested that experimentally derived δλ length extensions should be used as they are deemed to be more accurate than those provided via a Poisson simulation model.

In the next phase of the experiment, of interest was to examine if the length extensions from Equation 14, would hold when the permittivity was increased. To examine this, the outer diameter of the coaxial cell was designed such that it would provide a near perfect match when filled with a dielectric material. Calculations revealed the best permittivity for this test would only allow for a modest increase of ε_r_ = 2.5 or so, in order to preserve reasonable geometries for the inner and outer diameter of the coaxial cell. To accommodate this work, a dry sand was chosen for this phase of the experimentation, which was estimated by utilizing a network analyzer and the following techniques; free space through transmission, through transmission coaxial line, frequency domain reflectance network analysis of 20 cm TDR probe and finally standard TDR utilizing an HP 54120b with 12 GHz TDR reflectance head. The survey of all these techniques, for estimation of the sand’s true permittivity, produced a range of permittivity’s ranging from ε_r_ ≈ 2.4–3.2. Taking an estimate in the middle of the range, a sleeve was manufactured for the coaxial cell, such that with the sleeve removed, the coaxial cell provided one large outer diameter configuration, and with the sleeve inserted, provided a much smaller outer diameter that was designed to provide a matched condition when the cell was filled with the dry sand. Thus, the system was configured to avoid the miss-match impedance condition when the small outer diameter coaxial cell probe was filled with the dry sand, thereby providing an accurate estimate of the true permittivity of the dry sand as there would be minimal reflections allowing for a minimal error associated with the direct use of Equation 14 to estimate the sand’s permittivity, free from the influence of impedance miss-match errors.

In performing the experiment, the small OD coaxial cell was loaded with dry sand and the ¼ wavelength resonant frequency was found to be 1081 MHz, which with Equation 14, along with the experimentally derived δλ, obtained with air as the dielectric and application of Equation 15, provided an estimate of the permittivity of the sand to be ε_r_ = 2.87. For comparison, the ¼ wave frequency location as predicted for the large diameter coaxial cell with permittivity of 2.87 suggests the ¼ wave resonant frequency, with large cell’s δλ correction also provided by the air test, should occur at 1,024.1 MHz while experimental results were found to occur at 1031.3 MHz. This translates to an agreement between the large cell’s prediction of the ε_r_ = 2.91 compared to the smaller matched cell’s permittivity measurement of ε_r_ = 2.87. Conversely, without correction, the ¼ wave resonant frequency at the same ε′ = 2.87, is predicted to occur at 1164 MHz. Alternatively, using the measured resonance frequency of 1031 MHz along with the probe’s physical length of 3.8 cm, leads to the erroneous prediction of permittivity of ε′ = 3.65. Noting the densely packed cotton has a permittivity range from ε′ = 1.80 to ε′ = 2.00, and uncontaminated oil at a nominal permittivity of 2.35, it becomes clear that for applications with low permittivity materials and at frequencies where the probe length is limited, necessary to avoid radiation conditions, the correction for fringe capacitance is critical for obtaining accurate answers that are independent upon probe length.

## 4. Conclusions

The results of this research indicate that the hypothesis that fringe capacitance creates an artificial length extension to the coaxial cell’s center conductor is valid and is critically important for obtaining accurate measurements of permittivity in applications where the permittivity values are low and the length of the probes are limited due to the need to avoid placing the probe into radiation condition where the probe will effectively leak energy due to its length transition approaching the ¼ wave resonance location where it becomes a suitable antenna.

The magnitude of the fringe capacitance error, for a 3.8 cm probe, was found to cause a material with a permittivity of e’ = 2.87, to be incorrectly measured at e’ = 3.60 if the fringe capacitance was not accounted for. Furthermore we note that as the fringe capacitance acts as an effective probe lengthener, the error magnitude is dependent upon the probe length. As such, in transferring measurements from one laboratory to another that is likely using a different probe length, the only way to ensure transferability of the measurements is to account for the fringe capacitance.

The experimental results, and the Poisson model, suggest that this artificial length is not dependent upon permittivity but rather is a geometry factor and can therefore be used to correct and remove this artificial length once it’s been measured, regardless of the permittivity of the material. It is noted that the need for length correction is particularly important in drier soils and other media of low permittivity, such as cotton moisture sensing and water contamination of oils, and as the need for increased accuracy pushes the measurement into higher frequencies which limits the probe length. The technique outlined herein suggests a promising and simple method for quantifying an extra δγ pseudo correction length to account for the fringe capacitance.
